# An Improved Weighted Gradient Projection Method for Inverse Kinematics of Redundant Surgical Manipulators

**DOI:** 10.3390/s21217362

**Published:** 2021-11-05

**Authors:** Xinglei Zhang, Binghui Fan, Chuanjiang Wang, Xiaolin Cheng

**Affiliations:** 1 College of Mechanical and Electronic Engineering, Shandong University of Science and Technology, Qingdao 266590, China; zhangxinglei666@hotmail.com; 2 College of Mechanical and Electric Engineering, Zaozhuang University, Zaozhuang 277160, China; 3 College of Electrical and Automation Engineering, Shandong University of Science and Technology, Qingdao 266590, China; cxjwang@sdust.edu.cn; 4 College of Mechanical Engineering, Shandong University, Jinan 250100, China; chengxiaolin999@hotmail.com

**Keywords:** inverse kinematics, redundant surgical manipulator, joint position limits, kinematic singularity, improved weighted gradient projection method

## Abstract

Different from traditional redundant manipulators, the redundant manipulators used in the surgical environment require the end effector (EE) to have high pose (position and orientation) accuracy to ensure the smooth progress of the operation. When analyzing the inverse kinematics (IK) of traditional redundant manipulators, gradient-projection method (GPM) and weighted least-norm (WLN) method are commonly used methods to avoid joint position limits. However, for the traditional GPM and WLN method, when joints are close to their limits, they stop moving, which greatly reduces the accuracy of the IK solution. When robotic manipulators enter a singular region, although traditional damped least-squares (DLS) algorithms are used to handle singularities effectively, motion errors of the EE will be introduced. Furthermore, selecting singular region through trial and error may cause some joint velocities exceed their corresponding limits. More importantly, traditional DLS algorithms cannot guide robotic manipulators away from singular regions. Inspired by the merits of GPM, WLN, and DLS methods, an improved weighted gradient projection method (IWGPM) is proposed to solve the IK problem of redundant manipulators used in the surgical environment with avoiding joint position limits and singularities. The weighted matrix of the WLN method and the damping factor of the DLS algorithm have been improved, and a joint limit repulsive potential field function and singular repulsive potential field function belong to the null space are introduced to completely keep joints away from the damping interval and redundant manipulators away from the unsafe region. To verify the validity of the proposed IWGPM, simulations on a 7 degree of freedom (DOF) redundant manipulator used in laparoscopic surgery indicate that the proposed method can not only achieve higher accuracy IK solution but also avoid joint position limits and singularities effectively by comparing them with the results of the traditional GPM and WLN method, respectively. Furthermore, based on the proposed IWGPM, simulation tests in two cases show that joint position limits have a great impact on the orientation accuracy, and singular potential energy function has a great impact on the position accuracy.

## 1. Introduction

Compared with six degree of freedom (DOF) robotic manipulators, 7-DOF robotic manipulators not only ensure motion accuracy of the end-effector (EE), but also optimize other objectives due to the existence of a redundant DOF, such as avoiding obstacles and adapting to human action [1,2,3,4,5,6]. Therefore, redundant manipulators have been widely used in the medical and aerospace fields, etc.

However, 7-DOF manipulators not only improve the flexibility, but also increase the difficulty of solving the inverse kinematics (IK) solution [7]. Indeed, achieving the 6-DOF pose (position and orientation) given by the EE is equivalent to six equations and seven unknowns, and there are innumerable solutions in theory. However, only one set of the IK solution is needed in robot kinematics control. So far, many scholars have studied the IK solution of redundant manipulators. These methods include geometric method [8], gradient-projection method (GPM) [3,9], weighted least-norm (WLN) method [10], extended Jacobian matrix method [11], and analytical method [12], etc. Some artificial intelligence algorithms are also used in the IK solution of redundant manipulators, such as genetic algorithm [13], particle swarm optimization algorithm [14], neural network algorithm [15].

Geometric algorithms are not universal and their modeling and solving process is complex. The extended Jacobian matrix method can only obtain an approximate IK solution, which is not suitable for robotic manipulators with high accuracy. Analytical algorithm is only applicable to robotic manipulators with specific configurations, or robotic manipulators with some simplified processing [16,17]. It takes a long time for intelligent algorithms to solve IK, which is not conducive to real-time control.

The GPM and WLN methods are popular algorithms for solving IK of redundant manipulators, and have the effect of avoiding joint position limits. A weighted GPM [18] and a clamping WLN method [19] were proposed for IK of a 7-DOF manipulator. Due to the introduction of damping factors in the singular region, the position errors of the EE are as high as 96.3 mm and 25 mm, respectively, which are not suitable for robotic manipulators with high accuracy requirements. Hu [20] presented a gradient projection of a weighted Jacobian matrix method for IK of a planar 3-DOF manipulator. However, this method did not solve the problem of motion accuracy reduction caused by joint positions near their limits. Kelemen [21] introduced an IK algorithm to avoid joint position limits, singularities and obstacles. However, the position error of the EE is as high as 18 mm, and the orientation error is not analyzed. This is not conducive to the further operation of the EE. Jun [22] proposed an improved clamping WLN method for IK of a redundant manipulator. However, the constant value of the repulsive potential field would lead to the disadvantage of too large or too small repulsive force, which affected the motion accuracy of the EE.

When robotic manipulators move to a Jacobian singular configuration, the Jacobian inverse matrix becomes numerically unrealizable, which is not conducive to the motion control. In [23,24,25], a damped least-squares (DLS) algorithm was used to handle singularities. Although the introduction of damping factors can solve the shortcomings of singular configurations, the damping factors will lead to the decline of the motion accuracy of the EE.

The existing GPM and WLN methods do not completely keep redundant manipulators away from joint position limits and singular configurations, which can reduce the motion accuracy of the EE. Different from traditional redundant manipulator, the redundant manipulators used in the surgical environment require the EE to have high pose accuracy to ensure the smooth progress of the operation. Therefore, based on the advantages of GPM and WLN method, combined with the traditional DLS algorithm, an improved weighted GPM (IWGPM) which introduces a joint limit repulsive potential field function and singular repulsive potential field function as subtasks is proposed. A clamping weighted matrix can make joints stop at their limits, and the introduced joint limit repulsive potential field function generates virtual forces at the damping interval to make joints return to the flexible interval. In the unsafe region, virtual forces generated by the singular repulsive potential field function can make redundant manipulators move away from unsafe region and solve the singular problem thoroughly.

The remainder of this paper is organized as follows: The kinematics of redundant manipulators and the traditional GPM, WLN method, and DLS algorithm are briefly reviewed in Section 2. The IWGPM is proposed to solve the IK problem of redundant surgical manipulators with avoiding joint position limits and singularities in Section 3. In Section 4, to verify and evaluate the effectiveness of the proposed method, simulation results and discussion are performed. Finally, the conclusions are presented in Section 5.

## 2. Kinematics Formulations

The forward kinematics is a nonlinear function that describes the relation between the pose of the EE $x\in R^{m}$ and joint position $q\in R^{n}$ as follows:

(1) x =f(q)

The first-order derivation of the function:

(2) $$\dot{x}=J\dot{q}$$

describes the velocities from the joint to the EE, where $\dot{x}\in R^{m}$ represents the velocity vector of the EE, $\dot{q}\in R^{n}$ is the velocity vector of joints, and $J\in R^{m\times n}$ is a Jacobian matrix.

To obtain a unique solution $\dot{q}$, the Jacobian inverse $J^{-1}$ or pseudo inverse $J^{+}$ for non-redundant or redundant manipulators, respectively, is used, as follows [26]:

(3) $$\dot{q}=J^{-1}\dot{x}$$

for non-redundant manipulators (*m* = *n*) and:

(4) $$\dot{q}=J^{+}\dot{x}=J^{T}{\left({\mathrm{JJ}}^{T}\right)}^{-1}\dot{x}$$

for redundant manipulators (*m* < *n*).

It is not difficult to conclude that Equations (3) and (4) hold when $J^{-1}$ and $J^{+}$ exists, and Equation (4) provides a least-norm solution. When robotic manipulators approach a singular configuration, $J^{-1}$ or $J^{+}$ of the manipulators becomes numerically ill conditioned. Furthermore, the vicinity of singular configurations may also cause joint velocities to exceed the corresponding limits. To overcome this drawback, a DLS algorithm [23,25] is a widely used approach that sacrifices accuracy of the IK solution to generate an improved Jacobian matrix in the singular region. The DLS algorithm is expressed as:

(5) $$J^{T}\dot{x}=(J^{T}J+\lambda^{2}I_{m})\dot{q}$$

where $I_{m}\in R^{n}$ is an identity matrix, and λ is a damping factor.

For redundant manipulators, the self-motion can be realized with some subtasks, with not affecting the main task, which can be expressed by GPM [3]:

(6) $$\dot{q}=J^{+}\dot{x}+(I_{n}-J^{+}J)z$$

where $I_{n}-J^{+}J$ is the projection operator onto the null space of the matrix J, and *z* is a gradient vector of performance criterion as a subtask. The $(I_{n}-J^{+}J)z$ is the homogeneous solution for self-motion.

Avoidance for joint position limits as a subtask were presented as:

(7) $$H(q)=\frac{1}{n}\sum_{i=1}^{n}{\left(\frac{2q_{i}-q_{\mathrm{imax}}-q_{\mathrm{imin}}}{q_{\mathrm{imax}}-q_{\mathrm{imin}}}\right)}^{2}$$

where $q_{\mathrm{imin}}$ and $q_{\mathrm{imax}}$ are the lower and upper limits of the *i*-th joint position, and $q_{i}$ is the *i*-th joint position.

As a result, replace z with ∇H(q), Equation (6) can be derived further as follows:

(8) $$\dot{q}=J^{+}\dot{x}+k(I_{n}-J^{+}J)\nabla H(q)$$

where k is a positive scalar coefficient to make the gradient rate of H(q) to be minimized, and ∇H(q) is the gradient vector of H(q), which is described as:

(9) $$\nabla H(q)=\left[\begin{array}{cccc} \frac{\partial H(q)}{\partial q_{1}} & \frac{\partial H(q)}{\partial q_{2}} & \cdots & \frac{\partial H(q)}{\partial q_{n}} \end{array}\right]$$

The WLN method [10] is also used to avoid joint position limits through weighted factors, and the transformations are introduced as follows:

(10) $$J_{w}=JW^{-\frac{1}{2}}$$

(11) $${\dot{q}}_{w}=W^{\frac{1}{2}}\dot{q}$$

(12) $$\dot{q}=W^{\frac{1}{2}}J_{w}^{+}\dot{x}=W^{-1}J^{T}{\left({\mathrm{JW}}^{-1}J^{T}\right)}^{-1}\dot{x}$$

where $J_{w}$ and ${\dot{q}}_{w}$ are a weighted Jacobian matrix and a weighted joint velocity, respectively. The $W\in R^{n\times n}$ is a diagonal and positive weighted matrix, and the *i*-th diagonal element is expressed as:

(13) $$w_{i}(q_{i})=\{\begin{array}{c} 1+\left|\frac{\partial H^{*}(q)}{\partial q_{i}}\right|\begin{array}{cc} & \Delta \left|\frac{\partial H^{*}(q)}{\partial q_{i}}\right|\geq 0 \end{array}\\ 1\begin{array}{cc} & \Delta \left|\frac{\partial H^{*}(q)}{\partial q_{i}}\right|<0 \end{array} \end{array}$$

where $\Delta \left|\frac{\partial H^{*}(q)}{\partial q_{i}}\right|$ is the change rate of $\frac{\partial H^{*}(q)}{\partial q_{i}}$, and $H^{*}(q)$ is defined as follows:

(14) $$H^{*}(q)=\sum_{i=1}^{n}\frac{{\left(q_{\mathrm{imax}}-q_{\mathrm{imin}}\right)}^{2}}{4(q_{\mathrm{imax}}-q_{i})(q_{i}-q_{\mathrm{imin}})}$$

where $q_{\mathrm{imin}}$ and $q_{\mathrm{imax}}$ are the lower and upper limits of the *i*-th joint position, and *q_i_* is the *i*-th joint position.

**Figure 1:**
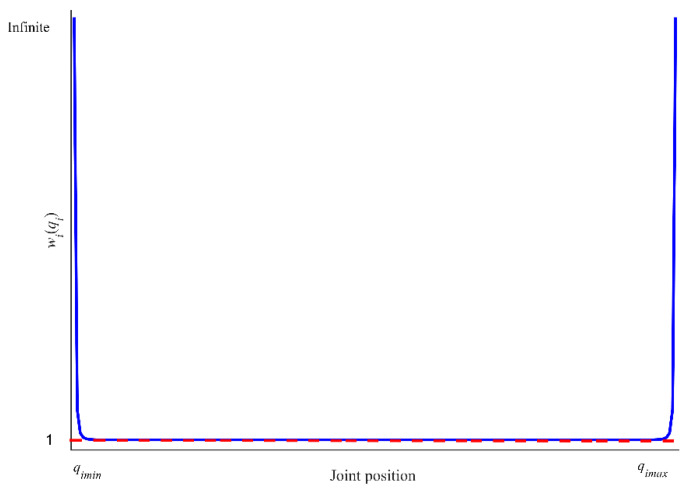
The change of $w_{i}(q)$ for the *i*-th joint position in WLN method.

Figure 1 shows the distributions of $w_{i}(q)$ with $q_{i}$. When $q_{i}$ is close to $q_{\mathrm{imin}}$ and $q_{\mathrm{imax}}$, $\left|\frac{\partial H^{*}(q)}{\partial q_{i}}\right|\to \infty$, this is $w_{i}(q)\to \infty$. From Equation (12), $q_{i}$ tends to zero, the corresponding joint stops at its limit position, but it is not far away from its limit.

Both GPM and WLN method can deal with singular problems, therefore, combining Equations (5) and (8) can be redefined by:

(15) $$\dot{q}=J_{G}^{+}\dot{x}+k\left(I_{n}-J_{G}^{+}J\right)\nabla H^{*}(q)$$

(16) $$J_{G}^{+}=J^{T}{\left({\mathrm{JJ}}^{T}+\lambda^{2}I_{m}\right)}^{-1}$$

Equation (12) can be rewritten as:

(17) $$\dot{q}=W^{\frac{1}{2}}J_{w}^{+}\dot{x}=W^{-1}J^{T}{\left({\mathrm{JW}}^{-1}J^{T}+\lambda^{2}I_{m}\right)}^{-1}\dot{x}$$

## 3. Improved Weighted Gradient Projection Method

The WLN method is much more efficient than the GPM one in avoiding joint position limits [20]. Due to the weighted matrix W, some joints stop at their limits, but this does not keep them away from their limits. Furthermore, on solving singular problems, DLS algorithms can prevent the problem of joint velocities exceeding the corresponding limits, but motion errors will be introduced. More importantly, DLS algorithms cannot guide redundant manipulators away from singular region. These conditions occur suddenly during the operation, which is very unfavorable to patients. Therefore, based on GPM, a joint limit repulsive potential field function and a singular repulsive potential field function are introduced in the null space to solve the shortages of GPM, WLN, and DLS methods.

### 3.1. Clamping Weighted Matrix and Joint Limit Repulsive Potential Field Function

**Figure 2:**
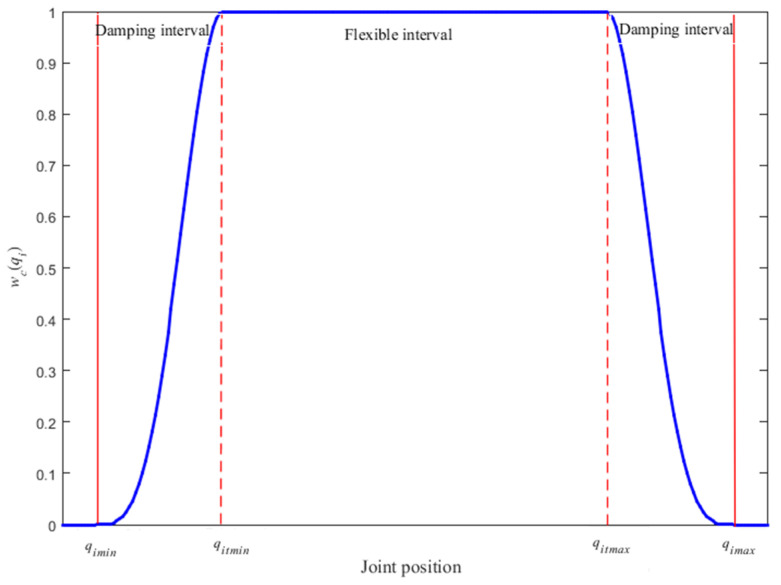
The change of $w_{c}(q_{i})$ for *i*-th joint position in clamping WLN method.

From Figure 1, it is easy to find that the starting and ending positions of W are difficult to determine, which sometimes affects the validity of the weighted matrix. Therefore, a buffer is added before the joint position limits, and the whole joint motion interval is divided into damping interval and flexible interval, as shown in Figure 2. That is a progressive clamping weighted matrix designed as follows:

(18) $$W_{c}=\mathrm{diag}(w_{c}(q_{i}))$$

(19) $$w_{c}(q_{i})=\{\begin{array}{cc} f(\frac{q_{\mathrm{itmin}}-q_{i}}{q_{\mathrm{itmin}}-q_{\mathrm{imin}}}) & q_{\mathrm{imin}}\leq q_{i}\leq q_{\mathrm{itmin}}\\ 1 & q_{\mathrm{itmin}}<q_{i}<q_{\mathrm{itmax}}\\ f(\frac{q_{i}-q_{\mathrm{itmax}}}{q_{\mathrm{imax}}-q_{\mathrm{itmax}}}) & q_{\mathrm{itmax}}\leq q_{i}\leq q_{\mathrm{imax}} \end{array}$$

where f(·) is a smooth function varying from 0 to 1. The $q_{\mathrm{itmin}}$ and $q_{\mathrm{itmax}}$ are the lower and upper damping thresholds before $q_{\mathrm{imin}}$ and $q_{\mathrm{imax}}$, respectively:

(20) $$f(x)\;=\;(-2x^{3}+3x^{2}{)}^{2}$$

(21) $$q_{\mathrm{itmin}}=q_{\mathrm{imin}}+\xi (q_{\mathrm{imax}}-q_{\mathrm{imin}})$$

(22) $$q_{\mathrm{itmax}}=q_{\mathrm{imax}}-\xi (q_{\mathrm{imax}}-q_{\mathrm{imin}})$$

where ξ is a positive constant scalar to determine the width of damping interval.

Then, Equation (17) can be rewritten as:

(23) $$\dot{q}=W_{c}J^{T}{\left({\mathrm{JW}}_{c}J^{T}+\lambda^{2}I_{m}\right)}^{-1}\dot{x}$$

When $q_{i}$ run in the flexible interval, $w_{c}(q_{i})=1$. While $q_{i}$ runs in the damping interval, the weighting matrix $w_{c}(q_{i})$ prevents $q_{i}$ from approaching its limit. When $w_{c}(q_{i})\to 0$, the corresponding joints stop moving forever, which will affect motion control and reduce pose accuracy of the EE.

A joint limit repulsive potential field function $\left(I_{n}-W_{c})R_{i}(q\right)$ is introduced to solve the drawback of Equation (23), and $I_{n}-W_{c}$ is also a smooth weighted matrix, when $q_{i}$ runs in the flexible interval, the joint limit repulsive potential field function is invalid. On the contrary, when $q_{i}$ starts to enter the damping interval, the joint limit repulsive potential field function $R_{i}(q)=\mathrm{diag}(r(q_{i}))$ comes into play to force the corresponding joints back the flexible interval as far as possible. The $r(q_{i})$ is defined as:

(24) $$r(q_{i})=\{\begin{array}{cc} \frac{q_{i}-q_{\mathrm{itmin}}}{q_{\mathrm{itmin}}-q_{\mathrm{imin}}}r_{\max} & q_{\mathrm{imin}}\leq q_{i}\leq q_{\mathrm{itmin}}\\ 0 & q_{\mathrm{itmin}}<q_{i}<q_{\mathrm{itmax}}\\ \frac{q_{i}-q_{\mathrm{itmax}}}{q_{\mathrm{imax}}-q_{\mathrm{itmax}}}r_{\max} & q_{\mathrm{itmax}}\leq q_{i}\leq q_{\mathrm{imax}} \end{array}$$

where $r_{\max}$ is the maximum potential field force, the closer the limit is, the bigger the potential field force. When $q_{i}$ goes towards $q_{\mathrm{imin}}$, $r(q_{i})$ is negative, and $r(q_{i})$ is positive when $q_{i}$ goes towards $q_{\mathrm{imax}}$.

Considering the joint limit repulsive potential field function and combining with GPM, Equation (23) becomes:

(25) $$\begin{array}{l} \dot{q}=W_{c}J^{T}{\left(JW_{c}J^{T}+\lambda^{2}I_{m}\right)}^{-1}\dot{x}\\ -(I_{n}-W_{c}J^{T}{\left(JW_{c}J^{T}+\lambda^{2}I_{m}\right)}^{-1}J)(I_{n}-W_{c})R_{i}(q) \end{array}$$

where the second part on the right-hand side is a homogeneous solution, which does not affect the main task.

### 3.2. A Novel DLS Method and Singular Repulsive Potential Field Function

In the traditional DLS algorithm, the damping factor function [27] is:

(26) $$\lambda_{\mathrm{traditional}}^{2}=\left\{ \begin{array}{ll} \lambda_{\max}^{2}\left(1-{\left(\frac{\sigma }{\sigma_{b}}\right)}^{2}\right) & 0\leq \sigma \leq \sigma_{b}\\ 0 & \sigma >\sigma_{b} \end{array}\right.$$

The $\varepsilon \in [0,\sigma_{b}]$ is the singular region, and the selection of $\sigma_{b}$ through trial and error may result in joint velocities exceeding the corresponding limits, so a novel damping function which includes a micro-buffer region $\bar{\varepsilon }\in \begin{array}{cc} (\sigma_{b}, & {\bar{\sigma }}_{b} \end{array}]$ is presented to avoid the joint maximum velocities being exceeded owing to the small selection of $\sigma_{b}$. The novel damping function is defined as:

(27) $$\lambda_{\mathrm{noval}}^{2}=\left\{ \begin{array}{ll} \lambda_{\max}^{2}\left(1-\;0.874\cdot {\left(\frac{\sigma }{\sigma_{b}}\right)}^{2}\right) & 0\leq \sigma \leq \sigma_{b}\\ \lambda_{\max}^{2}\left(0.5+0.5\cos\left(\frac{\pi \sigma }{{\bar{\sigma }}_{b}}\right)\right) & \sigma_{b}<\sigma \leq {\bar{\sigma }}_{b}\\ 0 & \sigma >{\bar{\sigma }}_{b} \end{array}\right.$$

(28) $${\bar{\sigma }}_{b}=\gamma \sigma_{b}$$

where γ is a positive constant scalar to determine the width of micro-buffer region. In this paper, the single region and micro-buffer region are called unsafe region.

**Figure 3:**
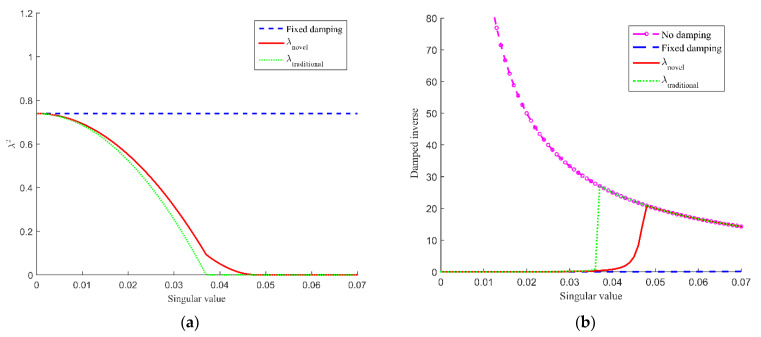
Distributions of damping factor (**a**) and damped inverse (**b**) with singular value, the example shows $\lambda_{\max}=0.86,\sigma_{b}=0.038,\gamma =1.3$.

The distributions of $\lambda^{2}$ and damped inverse $\frac{\sigma }{\sigma^{2}+\lambda^{2}}$ with singular value is depicted in Figure 3a,b, respectively. Through Figure 3b, it can be seen that in the case of no damping, infinite damping inverse will occur at a singular configuration which will result in very high joint velocities. Figure 3a,b show that the fixed damping value has the same effect in the unsafe region, which is unfair to the micro-buffer region, while the traditional damping function has an effect only in the singular region. However, except for the singular region, the new damping function still has a micro-damping factor in the micro-buffer to prevent the disadvantage that the joint speed exceeds the limit due to the small selection of $\sigma_{b}$. Furthermore, the damping continuity in the singular and micro-buffer regions ensures the continuity of joint velocities.

**Figure 4:**
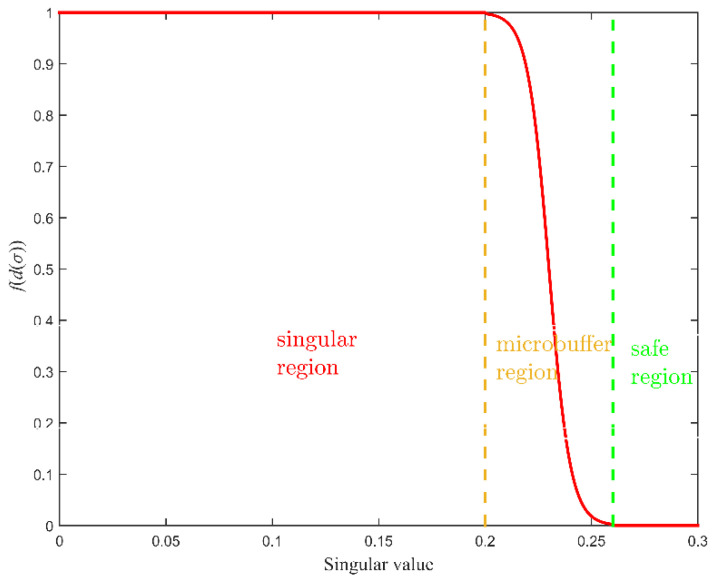
The shape of the function f(d(σ)) is set using two parameters: $\sigma_{b}$ and ${\bar{\sigma }}_{b}$. The example shows $\sigma_{b}=0.2$.

When solving singular problems, traditional DLS algorithms generate motion errors due to the introduction of damping factor in unsafe region, and it cannot guide robotic manipulators away from unsafe region. It is suitable for the manipulator with low accuracy requirements. However, this paper studies a surgical manipulator, which has very high requirements for pose. Therefore, a singular repulsive potential field function F(q) [28] is introduced to address the two deficiencies:

(29) $$F(q)=K\cdot \left[\frac{J\cdot \nabla d(q)}{\left\|\left|J\cdot \nabla d(q)\right|\right\|}\right]\cdot f(d\left(\sigma \right)|\sigma_{b},{\bar{\sigma }}_{b})$$

(30) $$f(d(\sigma )|\sigma_{b},{\bar{\sigma }}_{b})=\left\{ \begin{array}{ll} 1 & d(\sigma )<\sigma_{b}\\ \frac{1}{1+e^{\delta \cdot \left(d\left(\sigma \right)-\frac{\sigma_{b}+{\bar{\sigma }}_{b}}{2}\right)}} & \sigma_{b}\leq d(\sigma )\leq {\bar{\sigma }}_{b}\\ 0 & d(\sigma )>{\bar{\sigma }}_{b} \end{array}\right.$$

where K is a diagonal matrix that contains the maximum torque or force that we allow to be applied to the operator in each spatial dimension. The matrix ∇d(q) is used to direct the wrench away from unsafe region. The $f(d\left(\sigma \right)|\sigma_{b},{\bar{\sigma }}_{b})$ is given as [29], and d(σ) is the distance to $\sigma_{\min}=0$, the $\delta =12/\left({\bar{\sigma }}_{b}-\sigma_{b}\right)$ is recommended. It can be observed from Figure 4 that the change of f(d(σ)) in [$\begin{array}{cc} 0, & {\bar{\sigma }}_{b} \end{array}$] is smooth and continuous.

### 3.3. Resolution IWGPM

Considering 3.1 and 3.2, and combining with GPM, a novel method called IWGPM is defined as:

(31) $$\begin{array}{l} \dot{q}=W_{c}J^{T}{\left(JW_{c}J^{T}+\lambda_{\mathrm{noval}}^{2}I_{m}\right)}^{-1}\dot{x}(q)\\ -(I_{n}-W_{c}J^{T}{\left(JW_{c}J^{T}+\lambda_{\mathrm{noval}}^{2}I_{m}\right)}^{-1}J)(I_{n}-W_{c})R_{i}(q)\\ -(I_{n}-W_{c}J^{T}{\left({\mathrm{JW}}_{c}J^{T}+\lambda_{\mathrm{noval}}^{2}I_{m}\right)}^{-1}J)F(q) \end{array}$$

We define $J_{c}^{+}=W_{c}J^{T}{\left({\mathrm{JW}}_{c}J^{T}+\lambda_{\mathrm{noval}}^{2}I_{m}\right)}^{-1}$, the Equation (30) is represented as:

(32) $$\dot{q}=J_{c}^{+}\dot{x}(q)-(I_{n}-J_{c}^{+}J)(I_{n}-W_{c})R_{i}(q)-(I_{n}-J_{c}^{+}J)F(q)$$

In Equation (31), when $W_{c}=I_{n}$, Equation (25) becomes Equation (23). On the contrary, when $q_{i}$ is in the damping interval, $w_{c}(q_{i})$ decreases gradually from 1 to 0, and the corresponding joint velocity also gradually decreases to 0. Meanwhile, $r(q_{i})$ increases gradually from 0 to make $q_{i}$ return to flexible interval. In the safe region, all elements in F(q) are zero, indicating that the singular repulsive potential field function does not work. When redundant manipulators are gradually entering the micro-buffer region from the safe region, some elements that cause singularities in F(q) increase gradually from 0 to $F_{\max}$. In the singular region, the function F(q) is always $F_{\max}$. The function F(q) has pushed redundant manipulators away from the unsafe region. From Equation (32), it is found that $\left(I_{n}-J_{c}^{+}J)(I_{n}-W_{c})R_{i}(q\right)$ and $\left(I_{n}-J_{c}^{+}J)F(q\right)$ pick from the null space of $J_{c}$ do not generate any motion at the EE.

## 4. Simulation Results and Discussion

In this section, simulations in the paper are implemented with the aid of the MATLAB R2015a tool, and a computer equipped with an Intel Core™ i5-2450M CPU @ 2.50 GHz and 2 GB RAM as the control platform.

A path starts from an initial pose of the EE, $x_{\mathrm{initial}}$, to a desired pose, $x_{\mathrm{desired}}$. The path is divided into *M* smaller line segments for numerical integration. At any integration step, the joint rates required to move the EE are calculated. Joint velocities are integrated sequentially until the EE reaches the desired pose. Equations (8), (23), (25) and (32) can be realized by an iterative algorithm and further applied to IK solution of redundant manipulators. The iterative algorithm steps are as follows:

Step (1): Assume an initial pose expressed as Euler angles of the EE, $x_{\mathrm{initial}}\in R^{6\times 1}$.

Step (2): Plan a trajectory from $x_{\mathrm{initial}}$ to $x_{M+1}=x_{\mathrm{desired}}\in R^{6\times 1}$ with *M* intervals, and assume the running time of the motion, *T.*

Step (3): Calculate a planned velocity at the interval *k* that moves the EE toward $x_{\mathrm{desired}}$ as

(33) $$\dot{x}(k)=\beta \frac{(x_{\mathrm{desired}}-x(k))M}{(M+1-k)T}$$

where β>1 is a deceleration factor.

Step (4): Compute general equation $\dot{q}(k)=J^{+}(q(k))\dot{x}(k)$. Equations (8), (23), (25) and (32) can be substituted the general equation.

Step (5): Calculate q(k+1) through the following equation:

(34) $$q(k+1)=q(k)+\dot{q}(k)\frac{T}{M}$$

Step (6): Through Equation (1), the new pose of the EE is obtained.

(35) x(k+1)=f(q(k+1))

Step (7): Letting q(k)=q(k+1)

Step (8): If $x_{\mathrm{desired}}-x(k)\leq \rho$, where ρ is the accuracy of the actual pose and desired pose of the EE specified by the user.

Return;

Else;

Repeat steps 3 to 6 for k=1⋯M.

End.

To make the actual pose of the EE as close to the desired pose as possible, the closed-loop algorithm is used in the IK solution process [30]. Therefore, Equations (8), (23) and (32) can be represented as:

GPM:

(36) $$\dot{q}(k)=J^{+}(k)(\dot{x}(k)+\Lambda E_{e})+k(I_{n}-J^{+}(k)J(k))\nabla H(q(k))$$

WLN:

(37) $$\dot{q}(k)=W_{c}(k)J^{T}(k)(J(k)W_{c}(k)J^{T}(k)+\lambda^{2}I_{m}{)}^{-1}(\dot{x}(k)+\Lambda E_{e})$$

IWGPM:

(38) $$\begin{array}{l} \dot{q}(k)=J_{c}^{+}(k)(\dot{x}(k)+\Lambda E_{e})-(I_{n}-J_{c}^{+}(k)J(k))(I_{n}-W_{c}(k))R_{i}(q(k))\\ -(I_{n}-J_{c}^{+}(k)J(k))F(q(k)) \end{array}$$

where Λ is positive feedback gain, The $E_{e}\in R^{6\times 1}$ is tracking error between the desired pose and the actual pose of the EE, which is defined as follows:

(39) $$E_{e}={\left[\begin{array}{cccccc} E_{x}, & E_{y}, & E_{z}, & E_{\phi }, & E_{\theta }, & E_{\psi } \end{array}\right]}^{T}$$

where $E_{x}=p_{\mathrm{xdesired}}-p_{\mathrm{xactual}}$, $E_{y}=p_{\mathrm{ydesired}}-p_{\mathrm{yactual}}$, $E_{z}=p_{\mathrm{zdesired}}-p_{\mathrm{zactual}}$, $E_{\phi }=\phi_{\mathrm{desired}}-\phi_{\mathrm{actual}}$, $E_{\theta }=\theta_{\mathrm{desired}}-\theta_{\mathrm{actual}}$, $E_{\psi }=\psi_{\mathrm{desired}}-\psi_{\mathrm{actual}}$. The ($p_{\mathrm{xdesired}}$, $p_{\mathrm{ydesired}}$, $p_{\mathrm{zdesired}}$) and ($p_{\mathrm{xactual}}$, $p_{\mathrm{yactual}}$, $p_{\mathrm{zactual}}$) are desired position and actual position, respectively. The ($\phi_{\mathrm{desired}}$, $\theta_{\mathrm{desired}}$, $\psi_{\mathrm{desired}}$) and ($\phi_{\mathrm{actual}}$, $\theta_{\mathrm{actual}}$, $\psi_{\mathrm{actual}}$) are desired orientation and actual orientation expressed as Euler angles of the EE, respectively.

### 4.1. Case Study 1

**Figure 5:**
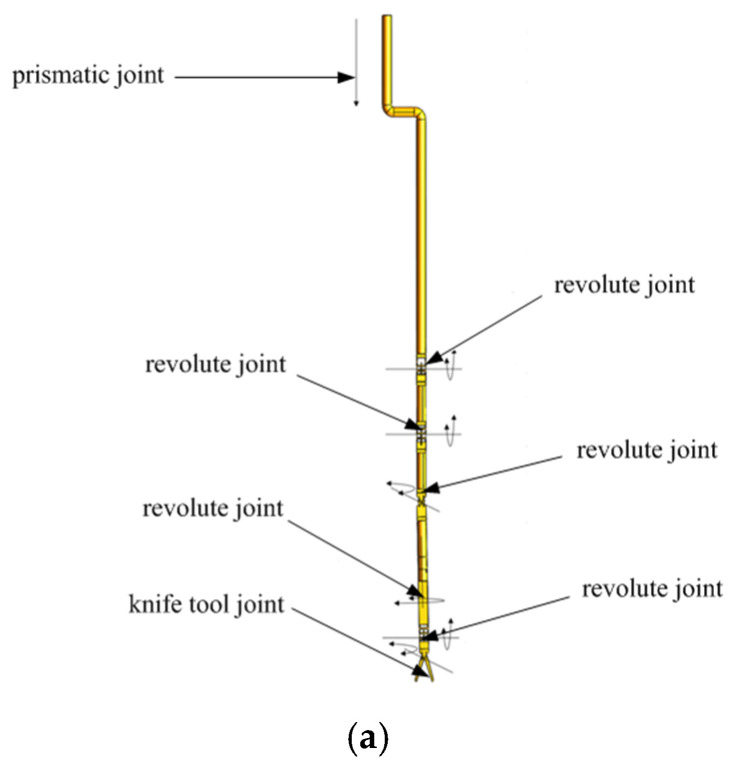

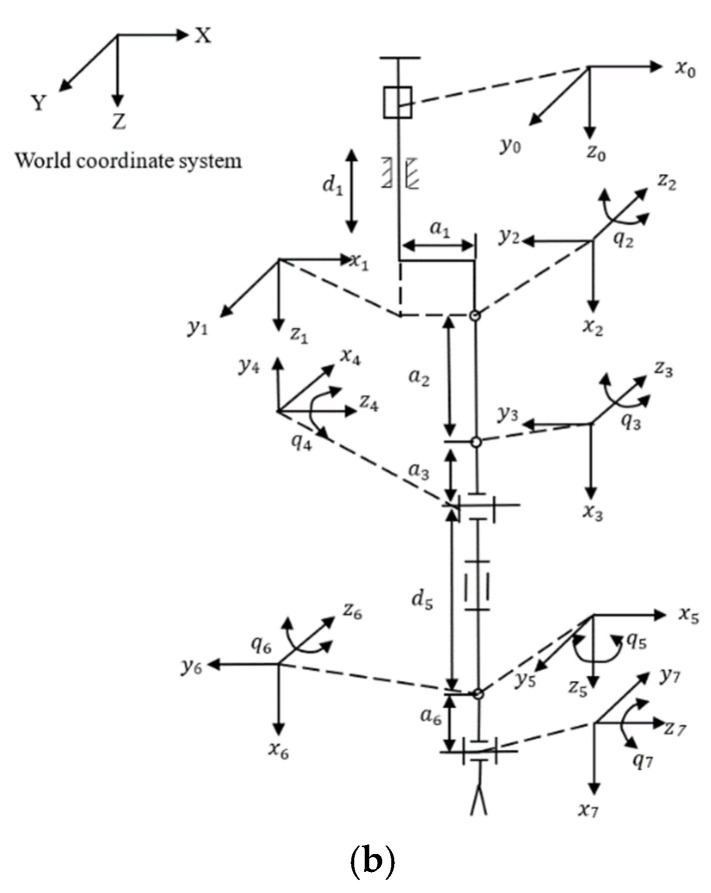
(**a**) The structure diagram of the 7-DOF manipulator; (**b**). The coordinate system of the 7-DOF manipulator.

Simulations are provided to demonstrate the validity and practicability of the proposed IWGPM method for IK solutions of the redundant manipulators. The self-developed 7-DOF surgical manipulator with a diameter of 10 mm is taken as the research object. Its structure diagram and coordinate system are shown in Figure 5a,b.

The DH parameters of manipulator with joint position limit range are shown in Table 1.

To illustrate the effect of the joint limit repulsive potential field function and singular repulsive potential field function, a straight-line path is selected as the trajectory. Moreover, the manipulator will enter a singular region, and some joints will be near their limit positions. The initial joint position $q_{\mathrm{initial}}=[44,\frac{\pi }{3},\frac{\pi }{6},\frac{\pi }{10},-1.4349,\frac{\pi }{4},\frac{\pi }{3}{]}^{T}$ and final joint position $q_{\mathrm{desired}}=[50,\frac{\pi }{5},\frac{\pi }{3},\frac{\pi }{6},\frac{\pi }{4},\frac{\pi }{3},\frac{\pi }{6}{]}^{T}$ of the manipulator is set (Note: The final joint position is used to calculate the desired pose of EE, which is to clarify that the joint position is different from that obtained by the proposed IWGPM.), respectively, which determines the initial pose and desired pose expressed as Euler angles of the EE:

$$p_{\mathrm{initial}}={[39.9883,\;117.4741,\;175.0739]}^{T}\left(\mathrm{mm}\right)$$

$$o_{\mathrm{initial}}={[0.7865,1.4431,-0.8411]}^{T}\left(\mathrm{rad}\right)$$

and the desired pose is:

$$p_{\mathrm{desired}}={[71.4062,\;106.7273,\;191.9349]}^{T}\left(\mathrm{mm}\right)$$

$$o_{\mathrm{desired}}={[-0.9057,1.2209,-0.0824]}^{T}\left(\mathrm{rad}\right)$$

For comparison, simulations with traditional GPM and WLN method are also presented. To reflect the coincidence degree of IK solution x and $x_{\mathrm{desired}}$, the pose accuracy is defined as follows:

(40) $$E=x_{\mathrm{desired}}-x_{\mathrm{final}}={\left[E_{x}^{*},E_{y}^{*},E_{z}^{*},E_{\phi }^{*},E_{\theta }^{*},E_{\psi }^{*}\right]}^{T}$$

where the $E_{x}^{*}$, $E_{y}^{*}$, and $E_{z}^{*}$ are the position errors in the *x*, *y*, and *z* directions between the final position and the desired position, respectively. The $E_{\phi }^{*}$, $E_{\theta }^{*}$, and $E_{\psi }^{*}$ is orientation error in ϕ, θ, and ψ directions between the final orientation and the desired orientation, respectively. The average deviation $E_{p}$ between the final position and the desired position, and the average deviation $E_{o}$ between the final orientation and the desired orientation are defined as follows:

(41) $$E_{p}=\frac{\left|E_{x}^{*}\right|+\left|E_{y}^{*}\right|+\left|E_{z}^{*}\right|}{3}$$

(42) $$E_{o}=\frac{\left|E_{\phi }^{*}\right|+\left|E_{\theta }^{*}\right|+\left|E_{\psi }^{*}\right|}{3}$$

To make simulations more convincing, in IWGPM, GPM, and WLN methods, we set $\lambda_{\max}=0.86$, ε=0.038, ξ=0.03, $r_{\max}=8$, β=2, $K={\left[\begin{array}{ccccccc} 0, & 0.08, & 0.08, & 0.08, & 0.08, & 0.08, & 0 \end{array}\right]}^{T}$, *T* = 10 s, *M =* 100, Λ=0.005.

#### 4.1.1. Simulation Analysis of the Proposed IWGPM

**Figure 6:**
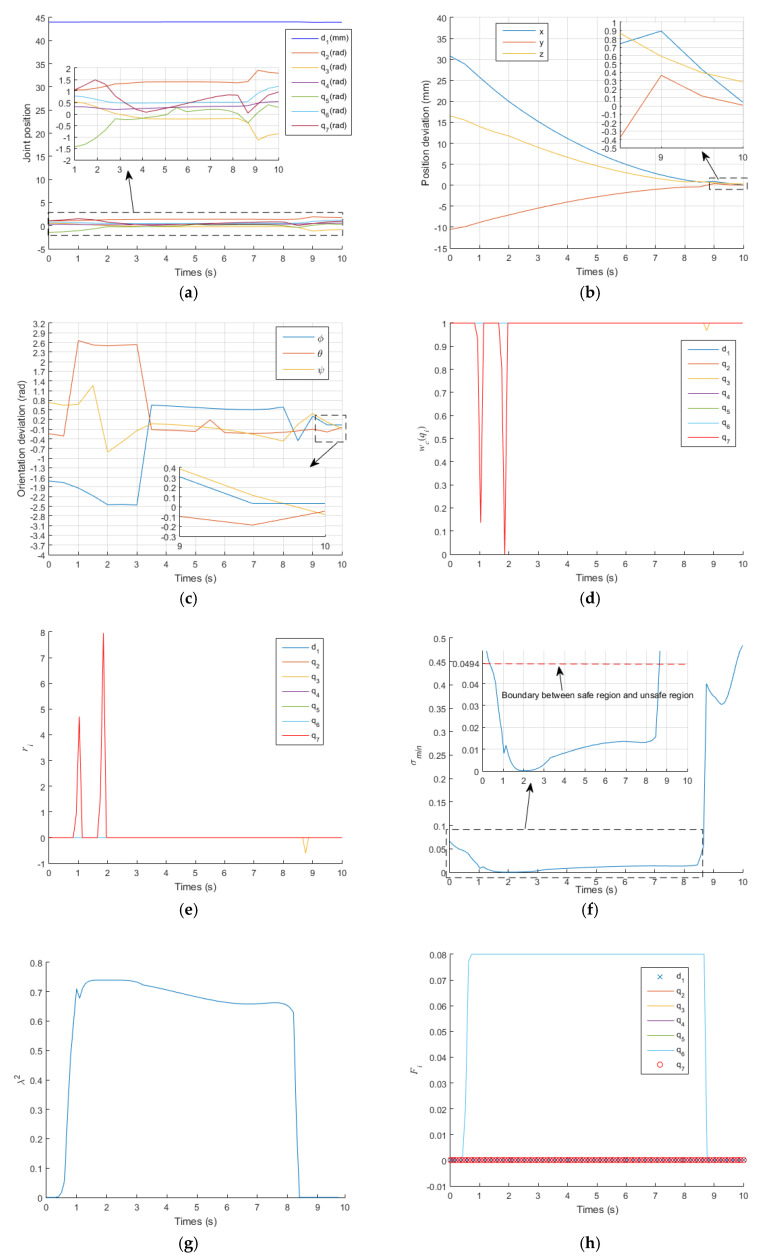

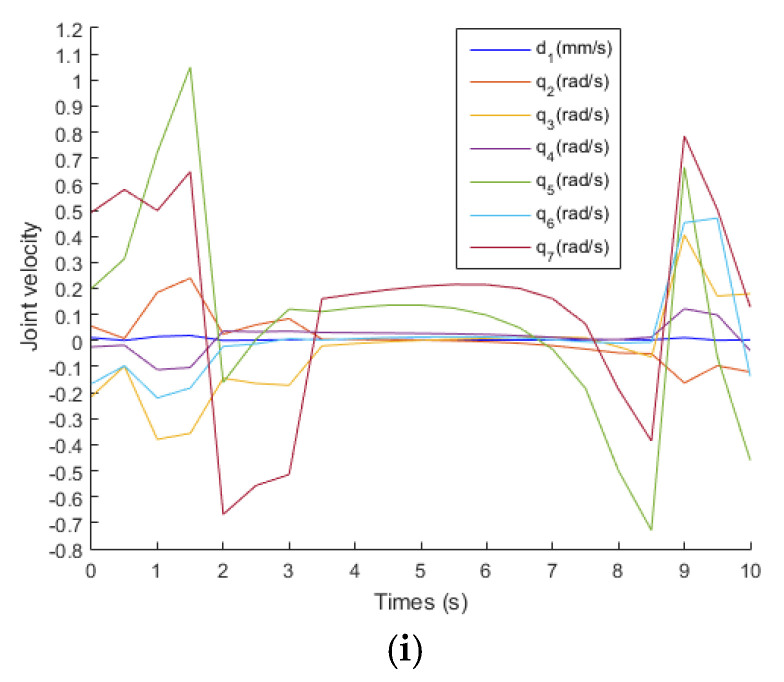
Simulations results of the proposed IWGPM. (**a**) the change curve of joint position; (**b**) the change curve of position deviation; (**c**) the change curve of orientation deviation; (**d**) the change curve of $w_{c}(q_{i})$; (**e**) the change curve of $r_{i}$; (**f**) the change curve of $\sigma_{\min}$; (**g**) the change curve of $\lambda^{2}$; (**h**) the change curve of $F_{i}$; (**i**) the change curve of joint velocity.

Figure 6 shows the results obtained with the proposed IWGPM, in which the IK solution is: $q_{\mathrm{IWGPM}}=[43.955,1.7786,-0.8667,0.5413,0.2849,1.2034,0.9499{]}^{T}$. The corresponding pose of the EE is $p_{\mathrm{IWGPM}}={[71.3652,\;106.7190,\;191.6448]}^{T}\left(\mathrm{mm}\right)$ and $o_{\mathrm{IWGPM}}={[-0.9381,1.2661,-0.0038]}^{T}\left(\mathrm{rad}\right)$, which are very close to $p_{\mathrm{desired}}={[71.4062,\;106.7273,\;191.9349]}^{T}\left(\mathrm{mm}\right)$ and $o_{\mathrm{desired}}={[-0.9057,1.2209,-0.0824]}^{T}\left(\mathrm{rad}\right)$. The average position deviation $E_{p}$ is 0.113mm and the average orientation deviation $E_{o}$ is 0.0387 rad. The pose accuracy of the EE is very high and meets the needs of surgery.

During the operation of the EE of the manipulator from the initial pose to the desired pose, Figure 6a shows the change curve of joint position. It is indicated that all joints operate within their position limits, and the position change curve of each joint shows continuity and small fluctuation. Figure 6b,c describe the deviation between the actual pose and the desired pose of the EE. In the initial stage, the actual pose is far from the desired pose. With the operation of time, it is more and more close to the desired pose. Figure 6d shows that $q_{7}$ is in the damping interval when t=1 s and t=1.8 s. Figure 6e indicates that the limit repulsive potential energy function of $q_{7}$ produces virtual forces $r(q_{7})=4.7111$ and $r(q_{7})=7.9569$ at the same time point, which makes $q_{7}$ enter the flexible interval. The joint $q_{3}$ is also in the damping interval when t=8.7 s. Figure 6e also indicates that the limit repulsive potential energy function of $q_{3}$ produces virtual forces $r(q_{3})=-6.066$ at the same time point, which makes $q_{3}$ enter the flexible interval. Figure 6f shows that the manipulator runs in the unsafe region from t=0.3 s to t=8.6 s, and the damping factor is generated during this time period to ensure that joint velocities operate within their limit, as shown in Figure 6g,i. Meanwhile, the singular potential energy repulsion function generates a virtual force $F(q_{2})=F(q_{3})=F(q_{4})=F(q_{5})=F(q_{6})=0.08$ to make the manipulator enter the safe region at t=8.7 s, as shown in Figure 6h.

#### 4.1.2. Simulation Analysis of the GPM and WLN Algorithm

**Figure 7:**
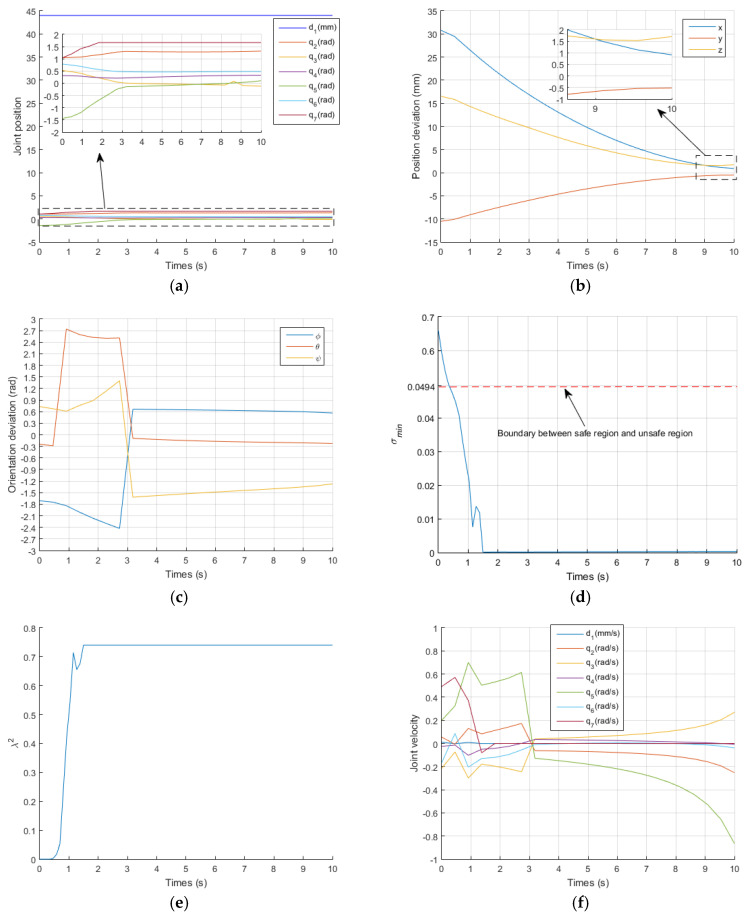
Simulation results obtained with the GPM. (**a**) the change curve of joint position; (**b**) the change curve of position deviation; (**c**) the change curve of orientation deviation; (**d**) the change curve of $\sigma_{\min}$; (**e**) the change curve of $\lambda^{2}$; (**f**) the change curve of joint velocity.

**Figure 8:**
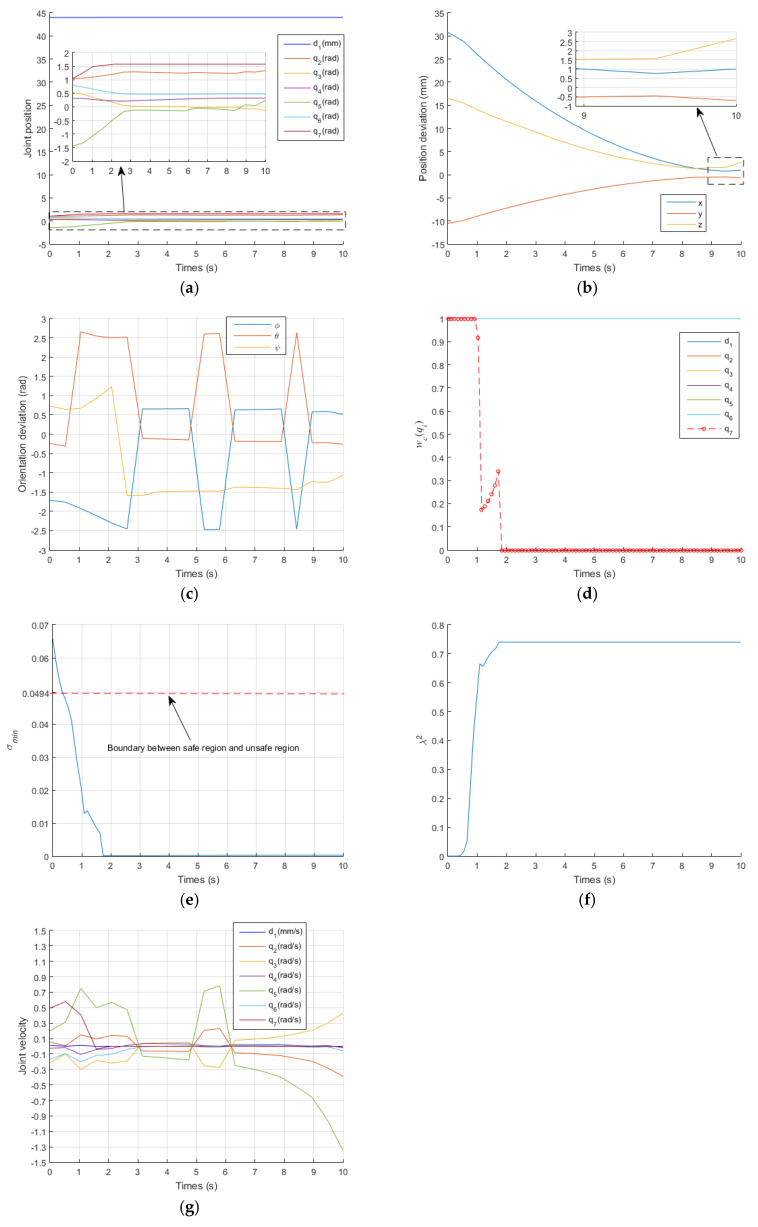
Simulation results obtained with the WLN method. (**a**) the change curve of joint position; (**b**) the change curve of position deviation; (**c**) the change curve of orientation deviation; (**d**) the change curve of $w_{c}(q_{i})$; (**e**) the change curve of $\sigma_{\min}$; (**f**) the change curve of $\lambda^{2}$; (**g**) the change curve of joint velocity.

Figure 7 and Figure 8 show the results of the traditional GPM and WLN method. In the two methods, $q_{7}$ exceeded its limit at t=2.1 s and t=1.8 s, respectively. It can also be clearly seen from Figure 8d that $q_{7}$ is in the damping interval from t=1s to t=1.8 s and exceeds $q_{7\max}$ from t=1.8 s to the end of operation. When a joint exceeds its position limit, the corresponding joint will stop moving. Furthermore, there is no virtual force to push the joint away from its limit position, so when $q_{7}$ exceeds its limit, it remains unchanged, as shown in Figure 7a and Figure 8a. It is known that the orientation of the manipulator is mainly controlled by the rear three joints. Therefore, the stop motion of $q_{7}$ seriously affects the orientation accuracy of the EE, as shown in Figure 7c and Figure 8c. Figure 7b and Figure 8b describe the deviation between the actual position and the desired position of the EE. In the initial stage, the actual position is far from the desired position. With the operation of time, it is more and more close to the desired position. According to Equations (41) and (42), for GPM, the average position deviation $E_{p}$ is 1.0452 mm and the average orientation deviation $E_{o}$ is 0.6058 rad. For WLN method, the average position deviation $E_{p}$ is 1.46 mm and the average orientation deviation $E_{o}$ is 0.6144 rad. It also shows that the position accuracy of the EE is acceptable, but the orientation accuracy is very low. Figure 7d and Figure 8e show that the manipulator runs in the unsafe region from t=0.5 s to t=10 s, and the damping value is generated during this time period to ensure that joint velocities operate within their limit, as shown in Figure 7e,f and Figure 8f,g, respectively.

In addition, Figure 7d and Figure 8e show that the manipulator is always in the unsafe region because of no singular potential energy function, while Figure 6f shows that the existence of singular potential energy function urges the manipulator to stay away from the unsafe region. i.e., the singular potential energy function introduced in this paper is very effective in solving singular problems.

Table 2 shows that the IK solution obtained by the proposed IWGPM. Compared with the proposed IWGPM, the position accuracy of the traditional GPM in *x*, *y* and *z* directions is reduced by 95.5%, 98.4%, and 82.9%, respectively. The orientation accuracy in ϕ, θ and ψ directions decreased by 94.3%, 97.7%, and 92.3%, respectively. The position accuracy of the traditional WLN in *x*, *y* and *z* directions is reduced by 96.0%, 98.8%, and 89.1%, respectively. The orientation accuracy in ϕ, θ and ψ directions decreased by 93.7%, 98.0%, and 92.6%, respectively. It can be seen that the proposed IWGPM can obtain very high pose accuracy, which fully meets the requirements of surgical accuracy.

### 4.2. Case Study 2

To observe the influence of joint position limits and singular potential energy function on the motion of the redundant manipulators. In this section, only the pose accuracy of the EE is considered for the simulation test in the following two cases. In case 1, based on the proposed IWGPM, joint position limit is removed and singular repulsive potential energy function is retained, Equation (38) is expressed as:

(43) $$\dot{q}\left(k\right)=J_{c}^{+}\left(k\right)(\dot{x}\left(k\right)+\Lambda E_{e})-(I_{n}-J_{c}^{+}\left(k\right)J\left(k\right))F(q\left(k\right))$$

In case 2, based on the proposed IWGPM, singular repulsive potential energy function is removed and joint position limit is retained, Equation (38) is presented as:

(44) $$\dot{q}(k)=J_{c}^{+}\left(k\right)(\dot{x}(k)+\Lambda E_{e})-(I_{n}-J_{c}^{+}(k)J(k))(I_{n}-W_{c}(k))R_{i}(q(k))$$

**Figure 9:**
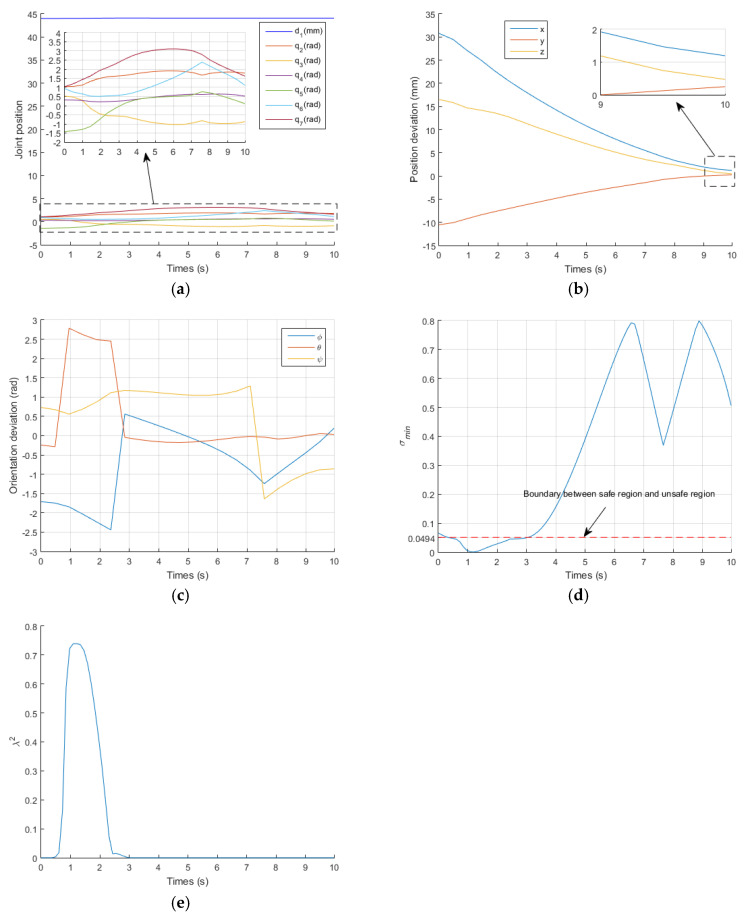
Simulation results of the proposed IWGPM (no joint position limits). (**a**) the change curve of joint position; (**b**) the change curve of position deviation; (**c**) the change curve of orientation deviation; (**d**) the change curve of $\sigma_{\min}$; (**e**) the change curve of $\lambda^{2}$.

**Figure 10:**
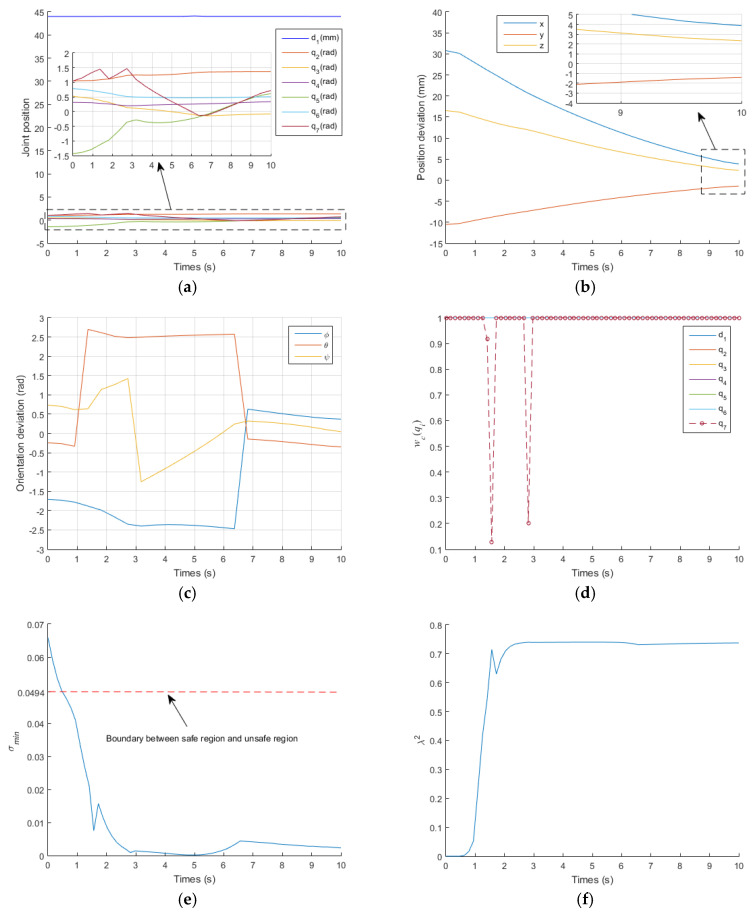
Simulation results of the proposed IWGPM (no singular potential energy function). (**a**) the change curve of joint position; (**b**) the change curve of position deviation; (**c**) the change curve of orientation deviation; (**d**) the change curve of $w_{c}(q_{i})$; (**e**) the change curve of $\sigma_{\min}$; (**f**) the change curve of $\lambda^{2}$.

The initial pose, desired pose of the EE and other parameter settings are the same as those in the previous section. The simulations are shown in Figure 9 and Figure 10. In the first case, the average position deviation of the EE is 0.6553 mm and the average orientation deviation is 0.3353 rad. Compared with the proposed IWGPM, the average position deviation is reduced by 82.8% and the average orientation deviation is reduced by 88.4%. In the second case, the average position deviation of the EE is 2.5319mm and the average orientation deviation is 0.2547 rad. Compared with the proposed IWGPM, the average position deviation is reduced by 95.5% and the average orientation deviation is reduced by 84.8%. It is not difficult to find that the position and orientation of the EE are affected differently in the two cases. The first case has a great impact on the orientation accuracy, and the second case has a great impact on the position accuracy.

In addition, Figure 9a shows that $q_{7}$ exceeds $q_{7\max}$ from t=1.1 s to t=10 s. However, Figure 10d shows that $q_{7}$ is in the damping interval at t=1.3 s, 1.6 s, 2.85 s. Meanwhile, the virtual force generated by the joint position potential energy function urges $q_{7}$ to enter the flexible interval, as shown in Figure 10a, which shows that the joint position potential energy function is very effective. Figure 10e,f show that the manipulator is always in the unsafe region from t=0.7 s to t=10 s. However, Figure 9d,e show that the manipulator after t=3 s is in the safe region, which shows that the singular potential energy function is very effective. From another point of view, this section verifies that the proposed IWGPM is very effective in solving joint position limits and singularity problems.

## 5. Conclusions

In this paper, the proposed IWGPM for the IK solution of redundant surgical manipulators is proposed to avoid joint position limits and singularities. The IWGPM introdues a clamping weighted matrix and joint limit repulsive potential field function. The clamping weighting matrix prevents approaching joint position limits, and the joint limit repulsive potential field function belong to the null space of weighted matrix only generates virtual force in the damping interval to make joints return to the flexible interval. Furthermore, a singular repulsive potential field function of belong to the null space of weighted matrix is introduced. When robotic manipulators enter an unsafe region, the singular repulsive potential field function produces an elastic force to drive manipulators back to the safe region. The proposed IWGPM is applied to the 7-DOF redundant surgical manipulator and compared with the traditional GPM and WLN methods. Simulations show that the proposed IWGPM is effective in avoiding joint position limits and singularities, and the pose accuracy is higher than achievable with the traditional GPM and WLN methods.

Furthermore, based on the proposed IWGPM, simulation tests in two cases are carried out. The results show that joint position limits have a great impact on the orientation accuracy, and singular potential energy function has a great impact on the position accuracy. Future work will focus on obstacle avoidance and optimal path planning of redundant surgical manipulators.

## Figures and Tables

**Table 1:** Modified DH parameters and joint position limits of the 7-DOF manipulator.

*i*	$\alpha_{i-1}\;(\mathrm{rad})$	$a_{i-1}\;(\mathrm{mm})$	$d_{i}\;(\mathrm{mm})$	$q_{i}\;(\mathrm{rad})$	$\left[q_{\mathrm{imin}},q_{\mathrm{imax}}\right]$
1	0	0	$d_{1}$	0	[−100,100] (mm)
2	π/2	20	0	$q_{2}$	[0,π] (rad)
3	0	68	0	$q_{3}$	[−π/2,π/2] (rad)
4	π/2	32	0	$q_{4}$	[0,π] (rad)
5	π/2	0	86	$q_{5}$	[−π,π] (rad)
6	π/2	0	0	$q_{6}$	[0,π] (rad)
7	π/2	20	0	$q_{7}$	[−π/2,π/2] (rad)

**Table 2:** The deviation of the EE between the actual pose and the desired pose at t=10 s.

Method	$E_{x}\;(\mathrm{mm})\;$	$E_{y}\;(\mathrm{mm})$	$E_{z}\;(\mathrm{mm})$	$$E_{\phi }\;(\mathrm{rad})$$	$E_{\theta }\;(\mathrm{rad})$	$E_{\psi }\;(\mathrm{rad})$
IWGPM	0.041	0.0083	0.2901	0.0324	−0.0052	−0.0786
GPM	0.9143	−0.5202	1.7011	0.5647	−0.2269	−1.0259
WLN	1.0137	−0.7137	2.6513	0.5159	0.259	−1.0682

## Data Availability

Not applicable.
